# A comparative two-cohort study of pediatric patients with long term stay in ICUs

**DOI:** 10.1038/s41598-021-84248-z

**Published:** 2021-02-25

**Authors:** Julia García Mancebo, Sara de la Mata Navazo, Estíbaliz López-Herce Arteta, Rosario Montero Mateo, Isabel María López Esteban, Adriana Mazzuchelli Domínguez, María Sánchez Doutel, Jesús López-Herce Cid, Rafael González Cortés

**Affiliations:** 1 Pediatric Intensive Care Unit, Gregorio Marañón General University Hospital, 2ª Planta Bloque D, Calle Doctor Castelo 47, 28007 Madrid, Spain; 2 Department of Maternal and Child and Public Health, Complutense University of Madrid, Plaza Ramón y Cajal S/N, 28040 Madrid, Spain; 3 Maternal and Child Health and Development Research Network RETICS Funded By Institute of Health Carlos III (ISCIII) Ref: RD16/0022, Madrid, Spain

**Keywords:** Paediatrics, Risk factors, Prognosis

## Abstract

During the last decades, the number of patients with long stay admissions (LSA) in PICU has increased. The purpose of this study was to identify factors associated with PICU LSA, assessing healthcare resources use and changes in the profile of these patients. A retrospective, observational, single-center study was carried out. Characteristics of LSA were compared between two periods (2006–2010 and 2011–2015). During the earlier period there were 2,118 admissions (3.9% of them LSA), whereas during the second period, there were 1,763 (5.4% of them LSA) (*p* = 0.025). LSA accounted for 33.7% PICU stay days during the first period and 46.7% during the second (*p* < 0.001). Higher use of non-invasive ventilation (80.2% vs. 37.8%, *p* = 0.001) and high-flow oxygen therapy (68.8% vs. 37.8%, *p* = 0.005) was observed in the 2011–2015 cohort, whereas the use of arterial catheter (77.1% vs. 92.6%, *p* = 0.005), continuous infusion of adrenaline (55.2% vs. 75.9%, *p* = 0.004), and hemoderivative transfusion (74% vs. 89.2%, *p* = 0.010) was less frequent. In the 2006–2010 cohort, hospital-acquired infections were more common (95.2% vs. 68.8%, *p* < 0.001) and mortality was higher (26.8% vs. 13.8%, *p* = 0.026). The number of long-stay PICU admissions have increased entailing an intensive use of healthcare resources. These patients have a high risk for complications and mortality.

## Introduction

Global child mortality has decreased over the last decades. Mortality reduction in patients admitted to pediatric intensive care units has resulted in an increase of patients with severe chronic disease and technology dependence. As a result of this, long-term intensive care stays are increasing. Some studies demonstrate that, as opposed to mortality associated with acute diseases, mortality in patients with chronic diseases has remained stable or has increased in the last decades^1,2^.

Although a standard definition remains elusive, a Pediatric Intensive Care Unit (PICU) stay exceeding 14 or 28 days is considered a long PICU stay^3^. Long-stay PICU patients account for a low percentage of PICU patients; however, these patients have been associated with a high mortality, morbidity, and resource consumption^4,5^. Several predictors of long PICU stay have been described, including dependence on mechanical ventilation; presence of comorbidities; a history of previous PICU stays; an age younger than 1 year; the administration of invasive techniques within 24 h of admission; or a high PRISM score at admission^5^.

Along with a high mortality, this group of patients also has a higher risk for complications such as hospital-acquired infections^6^; the need for a tracheostomy secondary to long-term use of mechanical ventilation^7^; or long-term physical and psychological problems following ICU treatment such as the so-called "post-PICU syndrome"^8^.

Early identification of patients at a higher risk for a long PICU stay may contribute to optimize critical care management. These patients could benefit from being assigned to specialized units. In the last years, several alternatives to conventional PICUs have been proposed including home care for technology-dependent patients or the creation of units for chronic or intermediate patients^9^. On the other hand, development of advance care plans could decrease futility in these patients^10,11^.

Despite the fact that some studies have previously defined the incidence and characteristics of patients with prolonged admission to the PICUs, changes in patients’ profiles and their number are possible over time. Therefore, it may be necessary to adapt healthcare resources and treatment strategies to improve medical care for a group of patients that seems to be growing.

Our hypothesis is that pediatric patients requiring long term admissions at PICUs are increasing in number and these patients have specific characteristics including high mortality and significant use of healthcare resources. Features and management of this group of patients may change with time. The primary objective of this study was to analyze the factors related to long-term PICU stay including use of healthcare resources and mortality in these patients. Secondly, to assess potential changes in the characteristics of long-stay patients by comparison of two historical cohorts.

## Materials and methods

An observational, retrospective, single-center study was conducted based on the review of the medical records of long-stay PICU patients.

A comparison of two historical cohorts was carried out. The first cohort included patients admitted between January 1st, 2006 and March 31st, 2010, whereas the second cohort included patients admitted between January 1st, 2011 and March 31st, 2015. The first cohort has been partially described elsewhere^12^. Patients were included in the study only if they were admitted between study dates of each period. Long stay patients who were admitted before the beginning of each study period were not included in the analysis although they stayed in the PICU during the study period. Those long stay patients who remained in PICU after the end of each study period were included in the analysis if their admission occurred during the study period. There is not a standardized definition for long-stay admissions and most studies stipulate a length of stay between 12 and 28 days. As in our previous study, long stay was defined as a length of stay ≥ 28 days^3,12^.

The study protocol was approved by the Gregorio Marañón General University Hospital Review Board. Obtention of informed consent was waived by the Gregorio Marañón General University Hospital Review Board. Data processing was performed in accordance with national laws and regulations.

### Study setting

The study was conduct in a PICU of a tertiary hospital. This is a 11-bed unit for patients from 1 month to 18 years old with both medical and surgical conditions. During the study period there was not intermediate care or high dependency unit in the hospital. Patients requiring non-invasive positive pressure ventilation or mechanical ventilation through tracheostomy, or hemodynamic support (including vasoactive drugs or ventricular assistance devices) were admitted only to PICU. Patients requiring high flow nasal cannula or requiring continuous infusion of sedation or analgesia could be admitted to a general hospitalization ward. This hospital is a national reference center for congenital heart disease providing surgical treatment and has both, a heart transplantation program and an extracorporeal life support program including extracorporeal membrane oxygenation (ECMO) and ventricular assistance devices.

### Study variables

Data were initially obtained from the PICU medical discharge reports and secondarily if any variable was not available from the electronic medical records (EMR). Data about the following variables were collected: age; sex; history of prematurity; presence of congenital abnormalities and heart disease; presence of cardiorespiratory arrest prior to admission; previous chronic respiratory disease; previous use of respiratory support at home; and baseline neurological status. A patient was considered to have history of prematurity when he was born before 37 weeks of gestational age. Baseline neurological status was determined as normal or altered if any degree of neurological impairment was described to be present before admission in the medical record. Data on the presence of cardiocirculatory, respiratory, renal, endocrine, metabolic, and hematologic alterations at admission were also collected from EMR. Cardiocirculatory alterations were considered to be present when the patient was hypotensive, tachycardic or needed vasoactive drugs at admission. Respiratory alterations were considered to be present when patient had hypoxemia, hypercapnia, increased work of breathing or needed invasive or non-invasive mechanical ventilation. Renal alterations were considered to be present if the patient was oliguria or had increased creatinine at admission. Metabolic or endocrine alterations were defined as present when the patients had acidosis or alkalosis or had electrolytic disturbances at admission. Hematologic alterations were considered in the presence of anemia, thrombopenia, leukopenia or hyperleukocytosis at admission. Other data retrieved included main reason for admission, length of PICU stay and whether the patient had been transferred from another hospitalization ward, the operating room, or directly from the emergency room.

In relation to the treatment administered and patient response, data were collected on the administration of intravenous sedation; type of respiratory support; need for vasoactive continuous infusion; use of renal replacement therapies; need for ECMO and/or use of a ventricular assist device; and use of other invasive techniques (central venous catheterization, arterial catheterization, tracheotomy, gastrostomy and bladder catheterization).

Mortality, incidence of hospital-acquired infection, cause of death, and therapy at the moment of death were also recorded. Hospital-acquired infections were considered only when clinical features were present and microbiological confirmation was obtained (by culture, PCR or any other result confirming presence of suspected causative microorganism). In survivors, discharge destination was also recorded.

### Data analysis

Data were entered into a database created for the purposes of the current study. Data analysis was performed using IBM SPSS Statistics v. 21.0 (IBM Corp. Armonk, NY) software package. Graphs were elaborated using DataGraph 4.5.1 (Visual Data Tools Inc, Chapel Hill, NC). Continuous variables without a normal distribution are expressed as median values and interquartile ranges (IQR) (p25–p75). Differences between normally-distributed continuous variables were assessed by Student's *t*-test, whereas comparison of continuous variables without normal distribution was carried out using non-parametric tests. Categorical variables were studied by the Chi square test. Fisher's exact test was used for variables involving less than five patients. The effect of different variables on mortality was assessed by multivariate logistic regression analysis adjusted for cardiac surgery as reason for admission, since a large proportion of our patients had undergone cardiac surgery, which could be a confounding factor in the study of mortality. The variables found to be associated with a significantly higher risk of mortality on univariate analysis were included in multivariate analysis. Linear regression was used to plot the trend of yearly admissions data. Data from years 2010 and 2015 was excluded from the linear adjustment as long stay patients were only included between January 1st and March 31st. A *p* < 0.05 was considered statistically significant.

## Results

**Figure 1:**
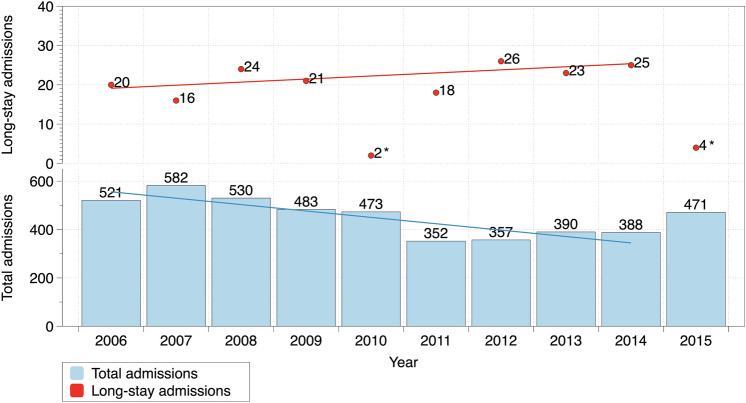
Total annual admissions and long-stay admissions during both study periods. Lines represent simple linear adjustment of yearly data. *During 2010 and 2015 long-stay admissions only include those long-admissions between January 1st and March 31st. Data from those years was excluded from the linear adjustment for both total annual admissions and long stay admissions. Figure elaborated using DataGraph 4.5.1 (Visual Data Tools Inc, Chapel Hill, NC).

During the earlier study period (2006–2010), there were a total of 2,118 admissions to the PICU, being 83 (3.9%) long stay admissions. During the later study period (2011–2015), there were 1,763 admissions to the PICU and 96 (5.4%) were long stay admissions (*p* = 0.025). Figure 1 shows annual number of admission and long stay admissions during the study periods. In total, in the first period patients with long stays accounted for 4,612 days of stay (33.7% of the total PICU stay days in the period) while in the second period long stay patients accounted for 6,388 days of stay (46.7% of the total PICU stay days), *p* < 0.001. There were 179 episodes of long stay that corresponded to 163 patients (16 patients had more than a long hospital stay).

Annual median length of stay for all admitted patients to the PICU during the first study period was 6.2 days (IQR 5.5–7) and 8.3 days (IQR 8.3–9.1) during the second study period (*p* = 0.008). The median of the length of stay in the first cohort of long-stay patients was 45 days (IQR 33–65) and 43 days in the second cohort (IQR 34–73) (*p* = 0.806). Table 1 displays the epidemiologic characteristics of patients with long-stay admissions of the two cohorts.

**Table 1:** Characteristics of the population of study.

	First cohort 2006–2010 (n = 83)	Second cohort 2011–2015 (n = 96)	*p*
Age, months. Median (IQR)	5 (3–38)	12 (4–42)	**0.044**
Sex (male)	54 (65.1%)	55 (57.3%)	0.288
Multiple admissions	12 (14.5%)	22 (22.9%)	0.150
Congenital malformations	68 (81.9%)	86 (89.6%)	0.141
Congenital heart disease	61 (73.5%)	80 (83.3%)	0.108
Only extracardiac malformations	7 (8.4%)	6 (6.3%)	0.574
Previous neurological deficit	6 (7.4%)	17 (17.7%)	**0.037**
Prematurity	11 (13.3%)	17 (17.7%)	0.413
Oncological disease	2 (2.4%)	2 (2.1%)	0.902
Previous tracheostomy	3 (3.6%)	4 (4.2%)	0.849
Previous gastrostomy	4 (4.8%)	14 (14.6%)	**0.030**
Heart transplant recipient	20 (24.1%)	18 (18.7%)	0.383

Statistically significant differences are shown in bold.

Each variable is expressed as the number of cases among the total number of patients in each cohort and percentage except for age which is expressed as mean and IQR.

*IQR* Interquartile rank.

**Figure 2:**
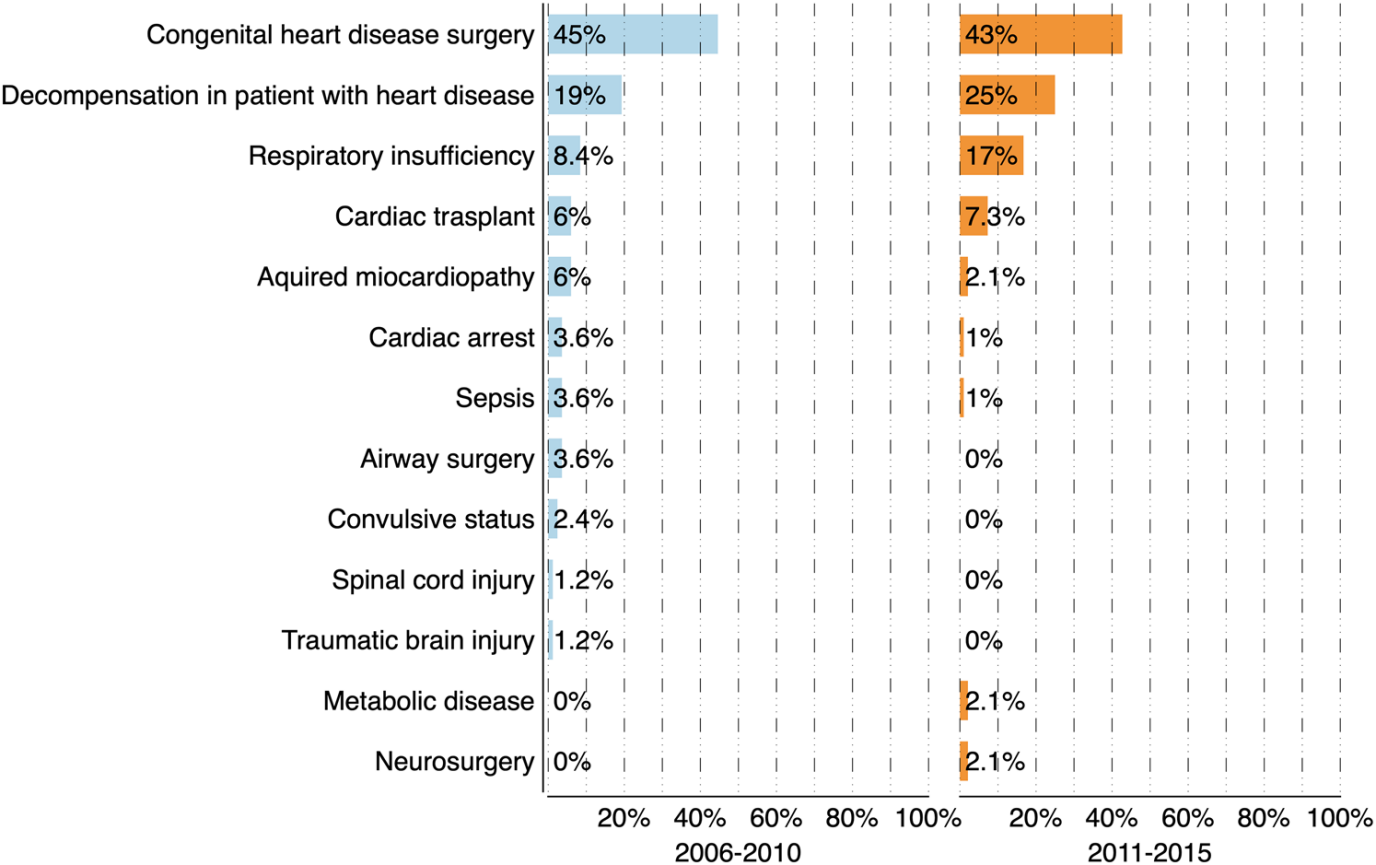
Reason for admission in the two cohorts of patients with long stay admissions. No statistically significant differences were observed between the two cohorts in any of the categories. Figure elaborated using DataGraph 4.5.1 (Visual Data Tools Inc, Chapel Hill, NC).

Figure 2 describes the main reason for admission in the two cohorts of PICU patients with long-stay admission. Eighteen patients (10.1%) had a cardiorespiratory arrest prior to PICU admission (8.4% in the first cohort vs. 11.5% in the second cohort, *p* = 0.502).

### Resources used

**Figure 3:**
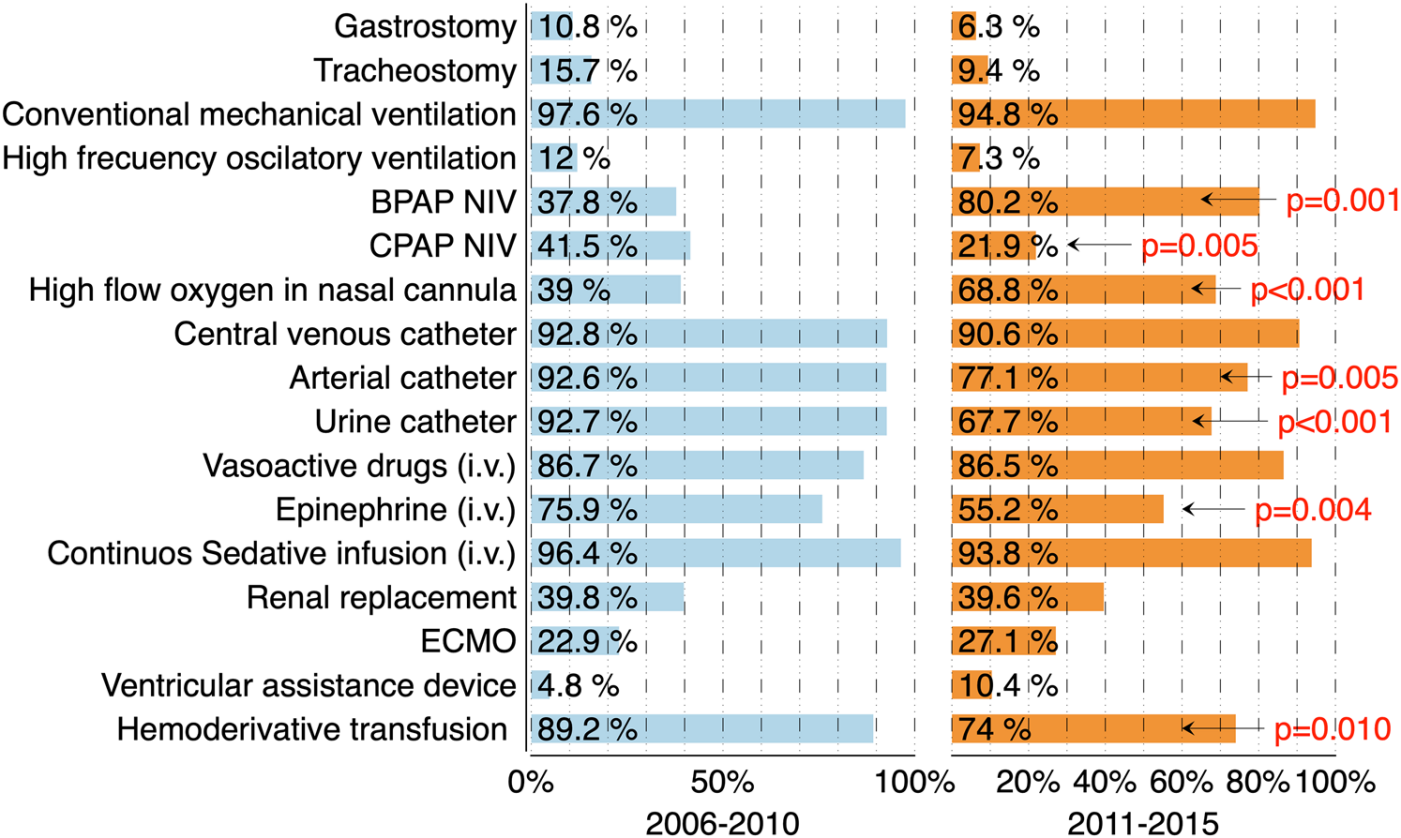
Resources used during PICU stay in the two cohorts of patients with long stay admissions. Statistically significant differences are pointed in the figure. Vasoactive drug use includes the continuous intravenous infusion of any vasoactive drug during the admission periods including epinephrine. CPAP NIV: Continuous positive airway pressure non-invasive ventilation. BPAP NIV: Bilevel Positive Airway Pressure Non-Invasive Ventilation. ECMO: Extracorporeal Membrane Oxygenation. Figure elaborated using DataGraph 4.5.1 (Visual Data Tools Inc, Chapel Hill, NC).

The healthcare resources used in long-stay PICU admissions are detailed in Fig. 3. There were no differences in the number of heart transplant recipients between the two cohorts.

### Complications during PICU stay

Laboratory-confirmed infections were present in 81% of total long stay admissions included in the study, with a significant reduction from 95.2% in the first cohort to 68.8% in the second (*p* < 0.001). The most frequent infections were pneumonia, 53.1% (71.1% in the first cohort vs. 37.5% in the second cohort *p* < 0.001); urinary infection (35.4% in the first cohort vs. 20.8% in the second cohort, *p* = 0.030). A reduction was also observed in catheter-associated infections (27.7% in the first cohort vs. 15.6% in the second, *p* = 0.049). No differences were found in the incidence of surgical wound infection, which was 11.9% for the two cohorts (*p* = 0.832). No significant differences were found either in the incidence of cardiac arrest (34.9% in the first cohort vs. 28.1% in the second, *p* = 0.327).

### Mortality

The overall mortality rate in the PICU was 2.6% (55/2118) during the first period versus 3.4% (60/1763) in the second (*p* = 0.144). The mortality of long-stay admissions accounted for 34.5% (19/55) of overall mortality in the first period versus 20% in the second period (12/60), *p* = 0.079.

The mortality of long-stay admissions was 22.9% (19/83) in the first cohort versus 12.5% (12/96) in the second, although this difference did not reach significance (*p* = 0.067). When patients with more than one long stay were excluded, mortality of long-stay PICU admissions was significantly higher in the first cohort (26.8%), as compared to the second (13.8%) (*p* = 0.026).

In the two cohorts, the most common causes of mortality were limitation of therapeutic effort and compliance with do not resuscitate orders (52.6% in the first cohort vs. 58.3% in the second) followed by causes in which resuscitation was attempted (36.9% and 25%) and death by neurologic criteria (10.5% and 16.7%), *p* = 0.756.

**Figure 4:**
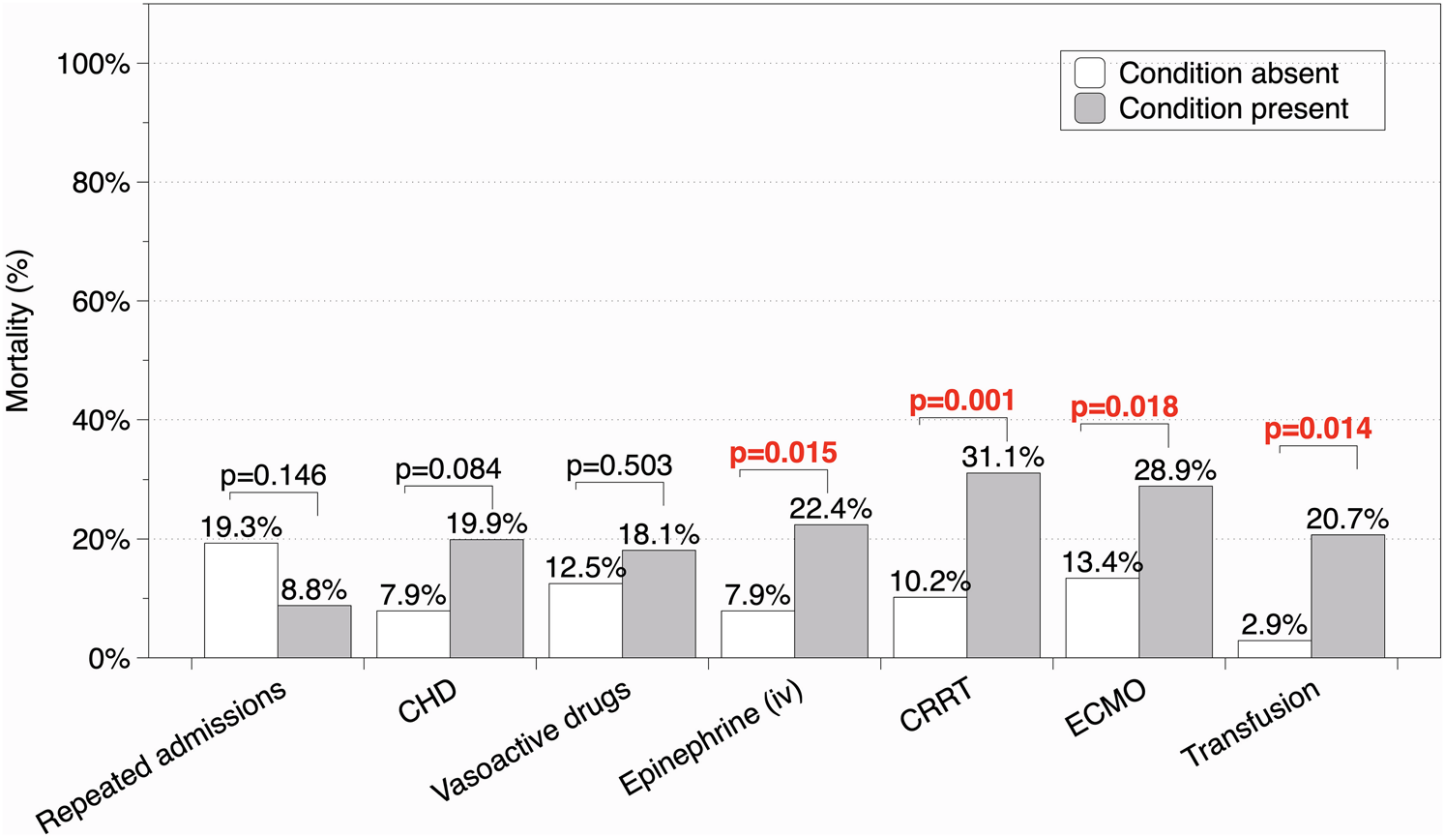
Mortality in relation to risk factors in the two cohorts (univariate analysis). CHD: Congenital heart disease. CRRT: Continuous renal replacement therapy. ECMO: Extracorporeal membrane oxygenation. Figure elaborated using DataGraph 4.5.1 (Visual Data Tools Inc, Chapel Hill, NC).

Univariate analysis demonstrated that mortality was higher among patients who received intravenous adrenaline infusion, renal replacement therapies, ECMO, and a hemoderivative transfusion (Fig. 4). Differences found to be significant on univariate analysis persisted in multivariate analysis after adjustment for cardiac surgery as main reason for admission (Table 2). Table 3 shows a comparative study of mortality between the two cohorts according to the presence of different risk factors.

**Table 2:** Multivariate analysis: mortality risk factors in long-stay admissions in the two study periods after adjustment for cardiac surgery as main reason for admission.

Adjusted for cardiac surgery as main reason for admission	OR (Mortality)	95% CI (OR)	*p*
ECMO	2.37	1.03–5.42	**0.041**
Hospital-acquired infection	8.08	1.06–61.89	**0.044**
Cardiac arrest during stay	68.46	15.11–310.08	** < 0.001**
Epinephrine (i.v.)	2.93	1.05–8.18	**0.040**
Renal Replacement therapy	5.39	2.21–13.10	** < 0.001**

*OR* Odds ratio, *CI* Confidence interval, *ECMO* Extracorporeal membrane oxygenation.

Statistically significant findings (*p*<0.05) are shown in bold.

**Table 3:** Comparative study of mortality between the two cohorts by factor of risk.

	First cohort (2006–2010)	Second cohort (2011–2015)	*p*
Previous heart disease	16/61 (26.2%)	12/80 (15%)	0.098
CHD correction	12/37 (32.4%)	6/41 (14.6%)	0.062
Congenital abnormalities	17/68 (25%)	11/86 (12.8%)	0.051
Only extracardiac malformations	1/7 (14.3%)	0/6 (0%)	0.938
CA prior to admission	3/7 (42.9%)	0/11 (0%)	**0.017**
Postoperative care admission	12/41 (29.3%)	6/54 (11.1%)	**0.025**
Heart transplant	5/20 (25%)	3/18 (16.7%)	0.529
ECMO	7/19 (36.8%)	6/26 (21.1%)	0.314
RRT	13/33 (39.4%)	10/38 (26.3%)	0.240

Each variable is expressed as the number of deaths among total admissions presenting each condition and percentage. *CHD* congenital heart disease, *CA* Cardiac arrest, *ECMO* Extracorporeal membrane oxygenation, *RRT* Renal replacement therapy.

Statistically significant findings (*p*<0.05) are shown in bold.

No differences were observed in discharge destinations after long-stay admissions between the two periods, being the hospitalization ward the most frequent discharge destination: 92.2% in the first and 91.6% in the second cohort.

## Discussion

Our study analyzes the characteristics of pediatric patients requiring long term admissions to PICU comparing two periods. Our study has found that despite the overall number of patients requiring long term admission is low, they account for more than a third of total stay days, and their proportion seems to be increasing. Most patients requiring long stays in PICU have specific clinical features such as congenital malformations (especially congenital heart disease) and they are at high risk of healthcare related infections and mortality.

There is not a standardized definition for long-stay admissions in the bibliography, with different length of stay cutoff points from 12^5^ to 28 days^13^. In our study, the 28-day definition was used as it corresponds with four natural weeks and implies a narrower definition of patients.

This study demonstrates that the proportion of long-stay PICU admissions is increasing, which is consistent with the findings of other authors. Although a low number of patients have a long stay in the PICU, long-stay patients have a considerable impact in terms of healthcare resource consumption and bed occupancy^5,7,9,13,14^. We have seen important variations in the global number of admissions during some years included in the study with a lower number of admissions in the second cohort. This finding might be related to changes in the numbers of available beds in the post-surgical recovery areas outside PICU.

Despite the decrease observed in the total number of admissions, the number of patients with prolonged admission seems to be increasing. Our study does not allow us to determine the causes of this rise in the number long-stay admissions to PICU. The observed decrease in mortality among patients with cardiac arrest prior to admission and patients admitted for postoperative care in the second cohort might partially explain this observation. Several other factors might be involved in this fact. Improvement in the treatment of acute patients, which would have died in the previous times can be pointed as one of the most important causes. Although, we have observed changes in the use of different modalities of respiratory support in long-term admitted patients, we cannot establish a direct relation of this with the increment of patients requiring long term admission. Beside this, changes in the attitudes of families and clinicians in the management of children with chronic pathologies and improvement of institutional support to their families might be also important reasons for this increment of long-stay patients. Healthcare systems should develop specific plans to address the increase in patients with long-term ICU admission.

In our study, long-stay patients were infants with congenital malformations, especially heart diseases. Although more than 80% of patients had congenital heart disease in both study periods approximately only half of them were admitted after surgery. This means that there was a high proportion of patients with congenital heart disease that were admitted for medical conditions. We have to point out that as our center is of national reference for congenital heart disease and heart transplant, some of these long stay patients were patients that required intensive care support while awaiting heart transplant. It is necessary that the centers in which patients with polymalformative syndromes and congenital heart disease are treated establish specific management plans for these patients, taking into account that they may require prolonged admissions to the PICU. A multidisciplinary approach to these patients seems essential in order to improve their prognosis.

The age at admission was older during the second period. As we considered that this could have been due to repeated admissions of the same patients in both periods as they grew older, only 4 patients were admitted in both periods with one admission in each period. We could not determine the reason for this increase with our data. The improvement of acute care of the most severe patients could lead to survival of patients which would not had survived previously.

### Consumption of healthcare resources

This study reveals that long PICU stay is associated with an intensive use of healthcare resources, which is consistent with previous studies^12,14^. However, some differences were observed between the two cohorts. This study showed a higher use of non-invasive mechanical ventilation and high-flow oxygen therapy, without a reduction in the use of invasive mechanical ventilation. This phenomenon could be due to the use of non-invasive ventilation in critical pediatric patients as a strategy for progressive respiratory support withdrawal to prevent reintubation in high-risk patients, as described by some authors^15,16^. Beside this, a higher use of non-invasive ventilation might be in relation with a lower (but not statistically significant) need of tracheostomy in the second cohort.

Some authors have documented that tracheostomy improves the quality of life of long-term mechanically-ventilated patients, reduces the need for analgesia and the rate of infections, being associated with a reduction of mortality rates^17,18^. In our study, tracheostomies placed prior or during admission were infrequent. This fact might reveal that reluctance to the placement of a tracheostomy in long-term mechanically-ventilated pediatric patients persists. Whereas early performance of a tracheostomy is commonplace in adults, the timing for the placement of a tracheostomy in pediatric patients has not yet been established and is usually performed at a later stage^7,17–20^.

The proportion of patients in which a gastrostomy was performed during their admission seemed to be higher in the first cohort (although there was no statistical signification), however the existence of a gastrostomy performed previously to admission was more frequent in the second cohort. Since the use of gastrostomy can serve to adequately meet their nutritional requirements, these findings might reflect changes in the strategies of nutritional support of complex patients. This is in line with the reports of other authors, who document a strong relationship between prognosis and the nutritional status of patients^21,22^ and the presence of different barriers to meeting their nutritional needs^23,24^. An appropriate nutritional support reduces the risk for complications and improves prognosis and the quality of life of long-stay patients, as it enhances their nutritional status^25^. However, as it occurred with tracheostomy, this procedure was only performed in a small number of patients.

It seems reasonable to consider that patients at risk of presenting prolonged admission are suitable candidates for the development of advanced care plans^10,11^.

Advance planning of the therapeutic strategies to be adopted in patients with prolonged hospitalization may be useful in reducing the use of healthcare resources, as well as the risks and costs related to admission. In the same sense, the establishment of advanced care plans can reduce the use of futile measures in these patients.

### Hospital-acquired infection

One of the most frequent complications in long-stay PICU patients are hospital-acquired infections. Rates between 10 and 20% of hospital-acquired infections have been documented in some series of long stay PICU patients^26^. The most common hospital-acquired infections in critically-ill pediatric patients include catheter-associated bacteremia followed by urinary tract infections (UTI) and ventilator-associated pneumonia (VAP)^6^. The data are in line with those obtained in our study and those described in the study by Miura et al., both including long stay patients^26^. We observed a significant reduction in the incidence of UTI, which may be related to the fact that the use of bladder catheters in these patients decreased by a third. A 50% reduction in the incidence of VAP was also documented. Although we cannot confirm that this was the case in our center, a higher adherence to recommendations for the prevention of VAP may have contributed to VAP incidence reduction^27,28^. A significant decrease was also observed in the rate of catheter-associated infections, which may be related to a series of factors including the use of chlorhexidine-impregnated dressings^29^, a lower use of arterial catheters, an increased use of peripherally-inserted central catheters or tunneled externalized catheters^30^, and the minimization of the time of use of central venous lines.

### PICU discharge

Most of our patients were discharged to other hospitalization wards, whereas only a small percentage of patients were directly discharged home. Long-stay patients are often technology-dependent or need specialized care, and the lack of specific care units may delay PICU discharge, thereby resulting in an increase in resource consumption. In the last decades, a more efficient use of healthcare resources has been achieved by the development of home care programs, which help overcome the many barriers to the discharge of technology-dependent patients as mechanically ventilated patients^31,32^. Another factor that has contributed to reduce healthcare resource consumption is the creation of intermediate care units or technology-dependent care units^33,34^. Our center has set up an intermediate care unit that was opened in 2019. One of the objectives of this unit is to facilitate the transition of technology-dependent patients to receive home care. Development of specific pediatric palliative care units has also been an important factor facilitating the discharge of long-stay PICU patients^35^.

### Mortality

Mortality was six times higher in long-stay patients as compared to other critically-ill pediatric patients, which is consistent with the findings of previous studies^36^. The results obtained in this study show a decrease of mortality in the second cohort, which may be associated with a lower incidence of hospital-acquired infections, which significantly increase morbidity and mortality in long-stay PICU patients. In agreement with other studies, the most common cause of death in our series was limitation of therapeutic effort^2,36^.

Early identification of patients at a higher risk for a poor evolution is crucial, as it will enable clinicians to adjust therapeutic strategies and establish advance care plans that avoid therapeutic obstinacy^37–39^. The patients that needed intravenous infusion of adrenaline, renal replacement therapies, ECMO, had a cardiorespiratory arrest or a hospital-acquired infection had a higher risk of mortality regardless of whether they were admitted after undergoing cardiac surgery. However, these indicators only reflect clinical severity but cannot be used to accurately predict outcomes in long-stay PICU patients in order to design an appropriate care plan.

### Limitations

The main limitation of this study is its retrospective design. Although this is a comparative study of two cohorts of long-stay PICU patients treated in two different periods, a comparative study was not performed with short-stay patients. Therefore, the factors found to be associated with higher mortality are only associated with long-stay patients. Our study does not include data on the long-term follow-up of the patients with prolonged admission. This information could be especially interesting in relation to the long-term functional outcomes, quality of life and deferred mortality of these patients. This is a single-center study performed in a PICU with a high proportion of patients with congenital heart diseases, and data cannot be generalized to PICUs with other types of patients. Additionally, the annual number of patients admitted to our unit makes it difficult to obtain a large sample size limiting the generalizability of the study results. For this reason, multicentric studies involving a more heterogeneous population are needed to obtain generalizable data on the characteristics and outcomes in long-stay PICU patients.

## Conclusions

In conclusion, there is a growing number of long stay patients, with a high incidence of congenital malformations, especially heart diseases, who have a long PICU stay, with the resulting high healthcare resource consumption. These pediatric patients have a high risk for complications and exhibit higher mortality rates as compared to short-stay patients. Early identification of PICU patients at a high risk of chronicity, the development of care programs that involve the family in the care of pediatric patients, along with the implementation of PICU adaptation measures and the creation of specific care units may improve the integral approach to these patients, as well as their prognosis and quality of life.

## Data Availability

Database used for the present study will be available upon reasonable request to corresponding author.
